# Correlative imaging using super‐resolution fluorescence microscopy and soft X‐ray tomography at cryogenic temperatures provides a new way to assess virosome solutions for vaccine development

**DOI:** 10.1111/jmi.13054

**Published:** 2021-09-03

**Authors:** Chidinma A. Okolo, Archana Jadhav, Patrick Phillips, Maud Dumoux, Amanda A. McMurray, Vishwas D. Joshi, Claire Pizzey, Maria Harkiolaki

**Affiliations:** ^1^ Beamline B24, Diamond Light Source, Harwell Science and Innovation Campus Didcot Oxfordshire UK; ^2^ Institute of Structural and Molecular Biology Rosalind Franklin Institute Fermi Avenue, Rutherford Appleton Laboratory, Harwell Science and Innovation Campus Didcot Oxfordshire OX11 0QS UK; ^3^ Activirosomes Limited, Centrum Norwich Research Park Norwich UK; ^4^ Seagull BioSolutions Private Limited Maharashtra India

**Keywords:** biomaterials, correlative microscopy, fluorescence, high resolution, vaccine, virosomes, X‐ray microscopy

## Abstract

Active virosomes (AVs) are derivatives of viruses, broadly similar to ‘parent’ pathogens, with an outer envelope that contains a bespoke genome coding for four to five viral proteins capable of eliciting an antigenic response. AVs are essentially novel vaccine formulations that present on their surface selected viral proteins as antigens. Once administered, they elicit an initial ‘anti‐viral’ immune response. AVs are also internalised by host cells where their cargo viral genes are used to express viral antigen(s) intracellularly. These can then be transported to the host cell surface resulting in a second wave of antigen exposure and a more potent immuno‐stimulation. A new 3D correlative microscopy approach is used here to provide a robust analytical method for characterisation of Zika‐ and Chikungunya‐derivatised AV populations including vesicle size distribution and variations in antigen loading. Manufactured batches were compared to assess the extent and nature of batch‐to‐batch variations. We also show preliminary results that verify antigen expression on the surface of host cells. We present here a reliable and efficient high‐resolution 3D imaging regime that allows the evaluation of the microstructure and biochemistry of novel vaccine formulations such as AVs.

## Lay Description

A novel combination of microscopies involving X‐ray and laser light has been developed at the correlative cryo‐imaging beamline B24 of the UK synchrotron which can be used to analyse across‐ and within‐batch variability of active virosome vaccine formulations. We use 3D fluorescence imaging to localise viral components within vaccine vesicles and soft X‐ray tomography to characterise sample variability and impact upon delivery to cells. Moreover, we offer the next step in automation of data processing and evaluation to further enable rapid assessment of exosome‐based vaccines.

Active virosome vaccines are suspensions of membrane‐bounded vesicles that carry antigens and genetic material from select viral pathogens. These elicit both an initial immune response through their introduction and a subsequent sustained antigenic potential via gene expression in host cells.

In this case, as in all novel vaccine formulations, rapid assessment and batch standardisation are of paramount importance for the medical community and the methods described here provide a robust way of quick and efficient assessment and validation of formulations during research and development and at the production stages.

## 1 INTRODUCTION

Virosomes were first developed as a chimera of unilamellar liposomes and viral envelope proteins^1^ and are currently used as vehicles for drugs, metabolites and vaccines deliveries.^2^, ^3^, ^4^, ^5^ Ab initio, virosomes were a blend of biomaterials‐ liposomes and viral components, devoid of genetic material from the parent virus, thus rendering them non‐replicable in vivo.^3^, ^6^ Typically, liposomes were reconstituted as virosomes by hydrophobic fusion of viral envelope proteins to liposomal membranes, thereby conferring them with the propensity for efficient receptor‐mediated cell binding, internalisation, cytosolic release and immunogenicity, thus simulating the modus operandi of viruses.^1^, ^7^ Essentially, virosomes are exosomes exported out of host cells. As vaccine tools, the surface antigens of exosomes are presented to their target's cell membrane, thereby activating immune cells and stimulating the release of inflammatory factors. In this way, exosomes modulate the immune responses of their target host cells.^8^, ^9^

Active virosomes (AVs) technology, a proprietary vaccine delivery technology,^10^ advances the field further by re‐engineering virosomes to assume ‘replicability’ in living systems.^11^, ^12^, ^13^ The AVs described herein have an outer envelope bearing content similarities to a ‘parent’ virus, but with an artificially designed genome coding for a selection of respective viral antigen genes.^10^ To achieve immunogenicity, these AVs are engineered to display selected antigens on their surface, hence, effectively stimulating a first ‘anti‐target antigen’ immune response. It is noteworthy that these AVs are not able to replicate and produce progeny AVs; rather, they use a heterologous replication machinery that is already packaged in them capable of producing additional target virus antigen. New viral proteins can then be produced and displayed long term on the cell surface; this second wave of antigen exposure prolonging and sustaining the immune response thus increasing the potency of these vaccine formulations. In addition, these AVs cannot replicate to produce progeny virions and so are inherently safe vaccine candidates.^10^

To meet safety standards, vaccines must be safe, specific and reproducible;^14^, ^15^, ^16^, ^17^ case in point is the covid‐19 pandemic emergency authorisation of vaccines for use which retained an emphasis on public safety.^18^, ^19^ Vaccine development stages (pipeline) include: exploratory research/discovery and development (R&D), pre‐clinical studies, phases I–IV of clinical trials, regulatory review and approval (licensure), pilot and scale‐up manufacturing, quality control and post marketing surveillance for adverse reactions.^20^ For this study, we provide novel tools and protocols towards the pre‐clinical and R&D stages.

Conventionally, at the exploratory and pre‐clinical stages, pH, proteomics via mass spectrometry, nuclear magnetic resonance (NMR) spectroscopy, ion exchange chromatography, differential scanning calorimetry, thermogravimetric analysis, elemental analysis, bicinchoninic acid test and enzyme linked immunoassay (ELISA) are all classical and advanced methods employed to‐date in characterising vaccines based on physicochemical parameters.^21^, ^22^, ^23^, ^24^, ^25^ These traditional vaccine profiling approaches however, do not provide high‐resolution data on native‐state biological structures of vaccines or host cells reacting to vaccine presence. Hence, in order to understand the action of AV interactions, it is essential to perform high‐throughput correlative 3D imaging of AVs under near‐physiological conditions. Fluorescence microscopy coupled to a nanometer resolution imaging method could provide such a tool. Electron microscopy (EM) could satisfy the need for biophysical or morphological characterisation via direct imaging^26^, ^27^, ^28^, ^29^, ^30^ but, despite its achievable resolution of 2–5 nm, it has limitations such as a reduced field of view (FOV), reliance on staining for contrast generation and limited beam penetration depth.^31^ An alternative which allows rapid data collection to 25 nm resolution is soft X‐ray tomography at cryogenic temperatures (cryoSXTs). CryoSXT involves imaging of biological samples in near‐native/physiological states using the preferential absorption of soft X‐rays by carbon‐dense biological structures at the water window (the spectral area between the k absorption edges of carbon and oxygen). At these energies, biological structures absorb light more than the media that surround them, hence appearing darker in projections and can therefore be tracked in 3D. Due to the high‐penetration depth of soft X‐rays, SXT can be used to image samples up to 12 microns thick and therefore does not require sectioning or chemical treatment of cells.^32^, ^33^ To ensure data fidelity all samples need to be vitrified through snap‐freezing. Vitrification of biological samples^34^ is the gold standard for sample preparation for SXT,^33^, ^35^ electron microscopy^36^ and fluorescence microscopy^33^, ^37^, ^38^ because it confers the following advantages: (a) cryopreservation of biological structures in their near‐native state without the need for chemical fixation, (b) protection of biological samples from radiation damage following exposure to soft X‐rays and (c) immobilisation of samples to capture a snapshot in time as well as preventing drift during data acquisition.

At the UK synchrotron correlative cryo‐imaging beamline B24, cryoSXT is an established imaging mode accessible to both academia and industry.^35^ To complement and further augment the capacity of cryoSXT, the beamline offers access to a newly developed 3D super resolution fluorescence microscope: cryo‐structured illumination microscopy (cryoSIM).^38^ Thus, fluorescently tagged features of interest within biological samples can be unambiguously correlated with ultrastructural features obtained by cryoSXT – a technique combination within the collective of correlative light/fluorescence and X‐ray tomography (CLXT). Beamline B24, at the UK synchrotron Diamond Light Source, is currently the only facility worldwide that offers a fully integrated correlative 3D cryo‐imaging scheme in super resolution fluorescence and cryoSXT. To date, cryoSXTs paired with cryoSIM have been used to investigate a number of diverse biological systems such as intracellular viral release pathways^33^ and molecular cytotoxicity vectors produced by cytotoxic T cells.^39^

We have tested CLXT as a viable complementary tool for high‐throughput characterisation of AVs vaccine formulations. This protocol can be adapted to accommodate exploratory R&D and pre‐clinical proof‐of‐concept studies and offers high‐throughput quality control during pilot and scale‐up manufacturing stages. CLXT at beamline B24, allowed the visualisation of the 3D ultrastructure and antigen loading of AV populations (with and without viral payload) as well as their morphology across batches. We focused on the characterisation of the microstructure of AVs as well as understanding the structural variations arising from batch manufacturing processes. We also studied the recovery of cells over a 2‐day period following exposure to AVs where we found indications of antigen expression on the surface of host cells.

As a result of the challenges presented to global health, the target virus antigens selected for this work were category B priority pathogens, Chikungunya and Zika viruses.^40^ Both are mosquito‐borne viruses endemic across tropical areas of the world but with recorded incidences in temperate climates also.^41^, ^42^ The resulting infections can be persistent and have severe implications to the long‐term quality of life for patients. To date, there is no drug treatment nor vaccine available for immunisation against these pathogens; therefore, the development and optimisation of appropriate vaccines remain an urgent requirement for world health.^41^, ^42^

Here, we present a novel CLXT approach as a complementary technique to existing means of characterisation which are based on physicochemical properties.

## 2 MATERIALS AND EXPERIMENTAL TECHNIQUES

### 2.1 Experimental workflow

**FIGURE 1:**
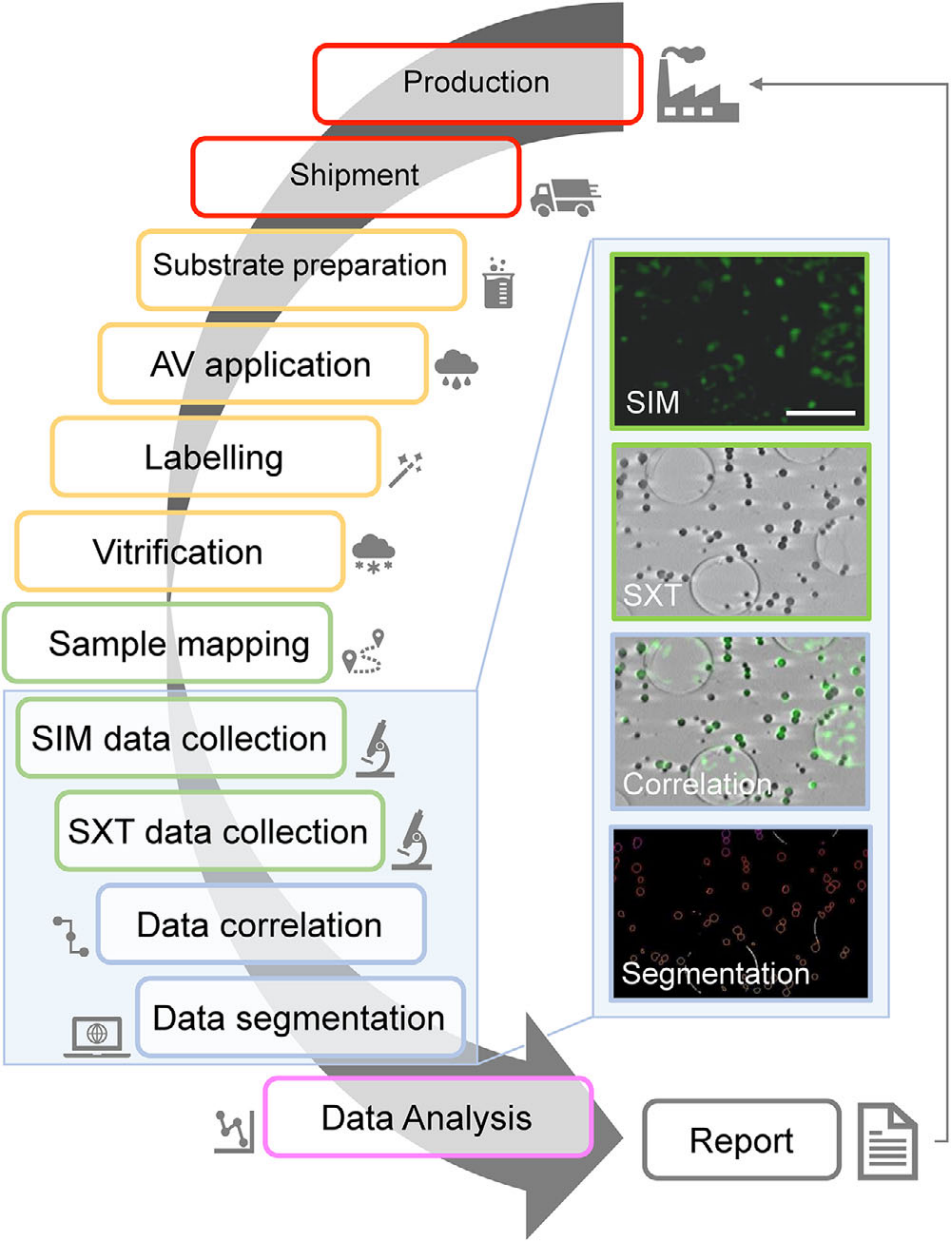
Flow chart of the work process at beamline B24 during vaccine characterisation studies. Representative AV sample SIM and SXT data can be seen in the panels to the right showing antigen specific intensity in green (SIM panel) and AV vesicle distribution in grayscale (SXT panel) with their overlay (Correlation) and the 3D segmented particles (bottom panel). Green fluorescence is generated here by immunolabelling of AV‐ZIKA‐2 antigens displayed on the AVs surface. Scale bar = 2 μm

The flowchart provided (Figure 1) delineates the workflow established workflow at beamline B24 for the characterisation of AV vaccine candidates. Manufactured AVs are shipped to B24 and either deposited on sample carriers (transmission electron microscopy 3.05 mm gold grids) or added to adherent cell populations. Samples are then fluorescently labelled at appropriate intervals using primary and tagged secondary antibodies for the viral antigens. They are also fiducialised to allow data alignment using gold nanoparticles before rapid freezing. Vitrified samples are first mapped with a conventional fluorescence microscope equipped with a cryo‐stage, then imaged by cryoSIM and then cryoSXT; data reconstruction for both imaging modalities happens in real time during data collection. Following successful data acquisition and reconstruction, datasets from both imaging platforms are correlated. The final stage of this pipeline involves the semi‐automated segmentation of AV particles and analysis to read‐out values for interpretation.

### 2.2 Generation of active virosomes

The eSAME technology employs reverse genetics on the measles virus genome (Seagull BioSolutions Private Ltd, Maharashtra, India). eSAME uses a helper plasmid that expresses the nucleocapsid protein, phosphoprotein and large RNA polymerase proteins (N, P & L) of measles alongside a virus‐coding plasmid that expresses the genomic RNA of the virus. Co‐transfection of these 2 plasmids is sufficient to produce complete virions. By replacing the virus‐coding plasmid with a cloning plasmid that contains multiple cloning sites with either coding or non‐coding sequences, non‐measles genes can be introduced.

AVs used here were produced by Seagull BioSolutions Private Ltd (Pune, Maharashtra, India) in partnership with Activirosomes Limited (Norwich, UK). AVs were generated by cloning the envelope glycoproteins (E1, E2 and E3) genes of Chikungunya and Zika viruses into the cloning plasmid (pMTXP1T); the plasmid vector was generated in the laboratory at Seagull BioSolutions Private Ltd (Pune, Maharashtra, India). The resulting plasmid is co‐transfected with a helper plasmid (pIRES‐N2aPL) into Vero‐M cells (a clone of Vero cells expressing the measles M protein). Once inside the cells the two plasmids re‐constitute the measles RNA dependent RNA polymerase (RdRP) system and express the E1, E2 and E3 proteins. The transfected cells thereafter serve as a production line for AVs. After 48 h, the culture medium is clarified by centrifugation at 5000 rpm for 5 min and the supernatant is subsequently concentrated by ultrafiltration through 300 kD filters.

## 3 SAMPLE PREPARATION AT B24

### 3.1 Tissue culture and AV application

AVs were sourced from different manufacturing batches and contained different virus antigens. Our naming convention follows the pattern AV‐ZIKA‐1 and AV‐CHIK‐1 for Zika virus and Chikungunya virus, respectively, with the final number referring to the manufactured batch that was supplied for this study (Table 1). AVs were shipped on dry ice and stored at −80°C pending use.

**TABLE 1:** Naming convention for AVs

Name	Active virosome	Batch
AV‐ZIKA‐1	Zika	1
AV‐ZIKA‐2	Zika	2
AV‐CHIK‐1	Chikungunya	1
AV‐CHIK‐2	Chikungunya	2

EM grids selected for this work were 3.05 mm gold transmission electron microscopy (TEM) grids (Quantifoil, Germany) that were incubated at 37°C in filtered serum overnight prior to sample addition to functionalise and increase hydrophilicity.^43^ AVs were added to Dulbecco's Modified Eagle Media (DMEM) with or without Vero cells at a ratio of 1:10, followed by layering on carbon‐coated EM grids prior to vitrification.

Vero cells were exposed to AV‐CHIK and AV‐ZIKA to assess incorporation, potency in cellular ultrastructure remodelling and cell surface expression of viral antigens. Following exposure, samples were plunge frozen at different time points corresponding to different times post‐exposure to AVs (*T* = 0 h, 1 h, 24 h, 48 h and 4 days).

### 3.2 Immunofluorescence labelling of AVs

AVs were immuno‐stained with fluorescently labelled secondary antibodies upon primary antibodies for Zika AVs or Chikungunya AVs. First, samples (cell or AV suspensions) were incubated for 5 min in primary antibodies: 1:500 of Zika virus envelope protein polyclonal antibody raised in rabbit (Insight Biotechnology Ltd) or 1:500 of anti‐Chikungunya virus antibody raised in sheep (NativeAntigen). Primary antibodies were diluted in DMEM supplemented with 10% foetal bovine serum (FBS) (Fisher Scientific, UK). The primary antibodies were washed off once with DMEM containing FBS for 2 min and then thrice with Hanks' Balanced Salt solution (HBSS) for 3 min each. The samples were further incubated for 15 min in secondary antibody solution: 1:5000 of sheep anti‐rabbit IgG conjugated to Fluorescin, FITC (Insight Biotechnology Ltd) against Zika AVs and 1:5000 of rabbit polyclonal antibody specific for sheep IgG, FITC (NativeAntigen) against Chikungunya AVs.

### 3.3 Preparation for cryo‐microscopy

Gold nanoparticles (BD Biosciences, UK) serve as fiducials during automatic data reconstruction of soft X‐ray tilt series into tomograms and for correlation of fluorescent and soft X‐ray images.^43^ Nanoparticles (provided by the manufacturer as 7 × 10^10^ particles/ml in dH_2_O/2 nM sodium azide) were prepared as a 1:10 dilution in serum‐free tissue culture medium and 2 μl were added to samples (AVs or cells). This was followed by 0.5–1 s blotting with Whatman filter paper No. 4 to reduce the overall depth of the sample. Samples were vitrified with a Leica EM GP2 (Leica Microsystems, Milton Keynes, UK) by rapidly cooling to temperatures below −170°C using liquid nitrogen‐cooled liquid ethane. Vitrified samples were stored under liquid nitrogen until needed for imaging

## 4 DATA ACQUISITION AND RECONSTRUCTION

### 4.1 CryoSIM data collection and reconstruction

**FIGURE 2:**
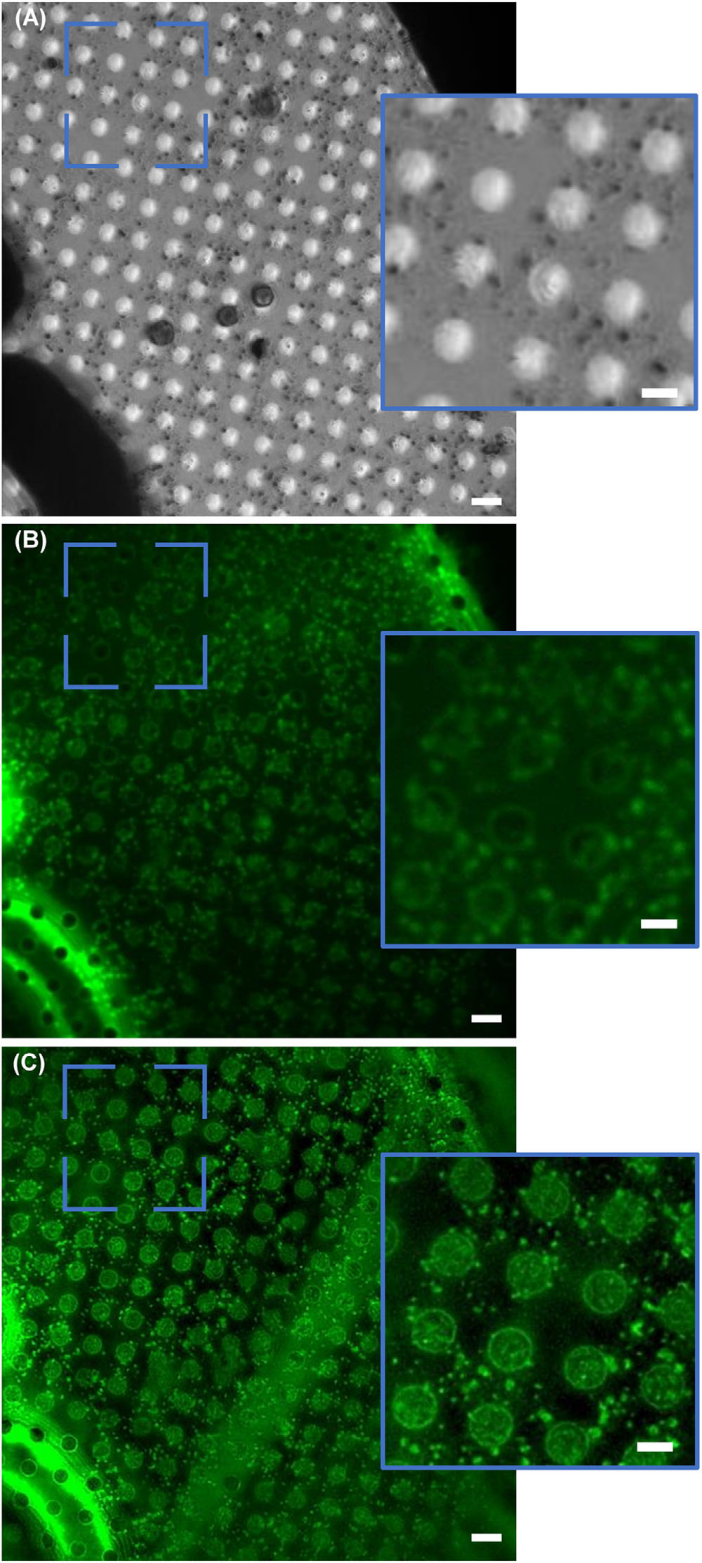
Representative AV data from the cryoSIM microscope at beamline B24. (A) Z projection (minimum intensity) of brightfield data of AV‐ZIKA‐2 using visible light on the cryoSIM. (B) A slice of 3D SIM AV‐ZIKA‐2 diffraction limited PWF data. (C) Slice of 3D SIM AV‐ZIKA‐2 reconstructed SIM data, showing obvious resolution gain. The green fluorescence signals denote tagged AV‐ZIKA‐2 proteins on the surface of AVs. Scale bars: (A)–(C) = 4 μm, insets = 2 μm

On the cryoSIM, a visible light mosaic (VLM) of each sample‐carrying EM grid was collected to identify areas of interest. For the AV samples, optimal fluorescence was achieved by exciting fluorophores with green laser light (488 nm wavelength) for 100 ms exposures time at 100 mW power, while collecting emitted light through a 525 nm filter on a conventional charge‐coupled device (CCD) camera at 3 MHz bandwidth. Bright field and structured illumination fluorescence data were acquired as a stack of images across the *Z* axis to provide 3D information (Figure 2A).^38^ To identify suitable grid positions for subsequent detailed cryoSXT measurements and to prevent destruction of fluorophores by soft X‐rays, cryoSIM measurements were always performed in advance of soft X‐ray measurements.^33^, ^38^

The data was reconstructed using SoftWoRx (GE Healthcare)^33^, ^38^, ^44^ (see Figure 2B, C for diffraction‐limited and SI reconstructed super resolution data examples). In cases where reconstruction failed in Fourier space due to vibrations during data collection, raw 3D SIM data were reconstructed into pseudo‐widefield (PWF) using the SIMcheck plugin in Fiji.^45^

### 4.2 CryoSXT data collection and reconstruction

**FIGURE 3:**
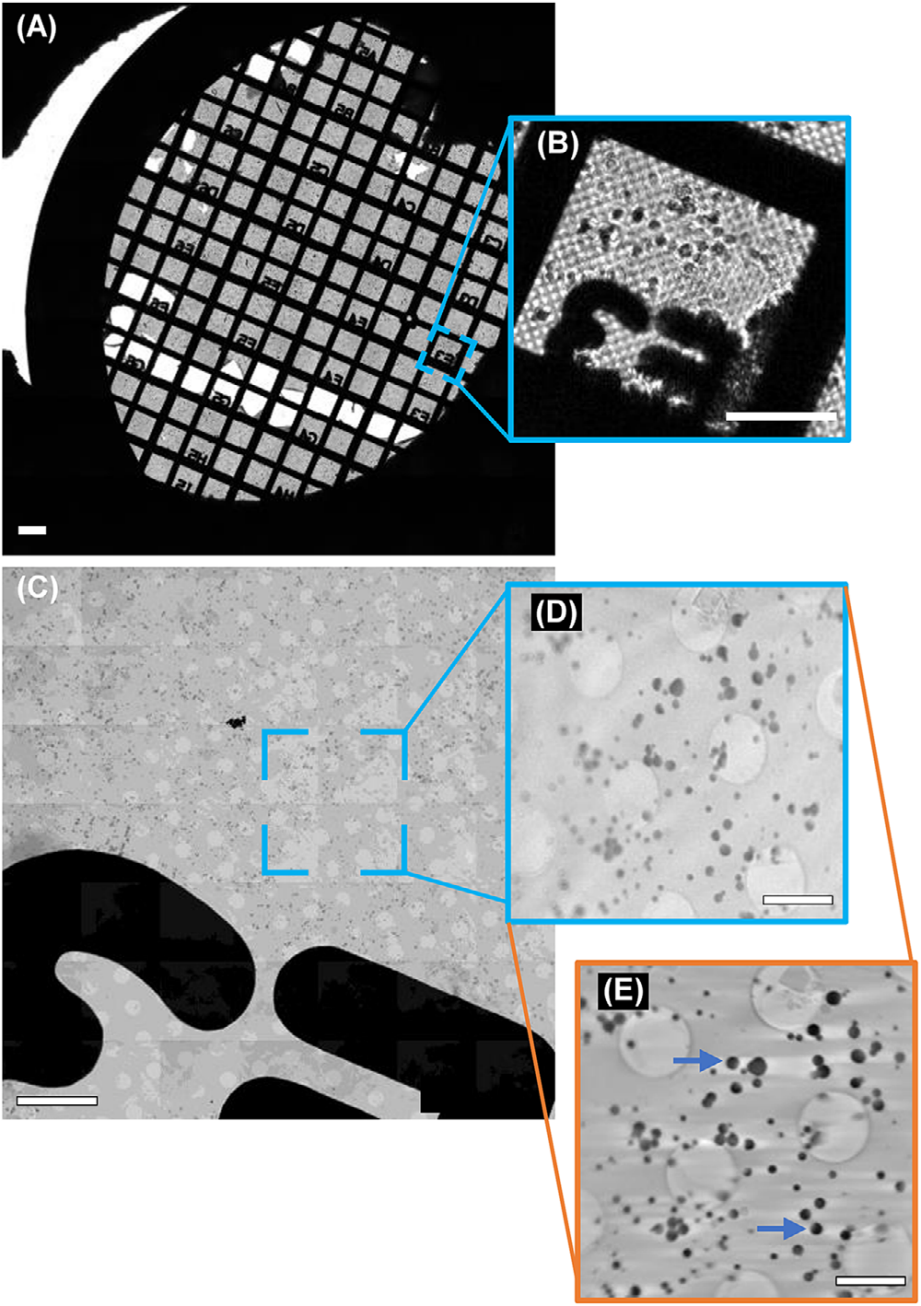
AV‐ZIKA‐2 data from cryoSXT (A) 2D mosaic of the sample grid using visible light. (B) Inset of the area highlighted in (A). (C) 2D overview X‐ray mosaic of the region highlighted in (A). (D) An X‐ray projection from a tilt series during raw data acquisition from the area highlighted in (C). (E) A slice from the 3D tomogram reconstruction from the same area in (C). The blue arrows are pointing at some of the AV‐ZIKA‐2. Scale bars: (A) 70 μm, (B) 35 μm, (C) 10 μm, (D) and (E) 2 μm

Vitrified samples were transferred with a transfer chamber to the transmission soft X‐ray microscope for SXT data collection. Low‐resolution visible light mosaics (Figure 3A,B) were created as the first step of the process to check the alignment of the whole grid and to locate the regions of interest, as identified by cryoSIM. Higher‐resolution 25 or 40 nm 2D X‐ray mosaics (Figure 3C) were then collected on regions of interests; key areas were identified and aligned with respect to the sample's centre of rotation and respective optics focal positions. For 3D visualisation of the AVs microstructure and cellular ultrastructure, tilt series (Figure 3D) were collected over an angular range, with maximum possible rotation from −70° to +70° (depending on the zone plate in use), with a step size of 1° or 0.5° and an exposure time of 2 s per frame.^33^, ^46^

Following data collection with soft X‐rays, tilt series were either manually reconstructed into 3D tomographic volumes (Figure 3E) with IMOD^©^ (University of Colorado)^47^ or an automated pipeline based on IMOD (batchruntomo) using simultaneous iterative reconstruction technique.^48^

## 5 POST‐DATA COLLECTION: IN SILICO PROCESSING AND ANALYSES

### 5.1 Correlation of fluorescence and X‐ray absorption data

**FIGURE 4:**
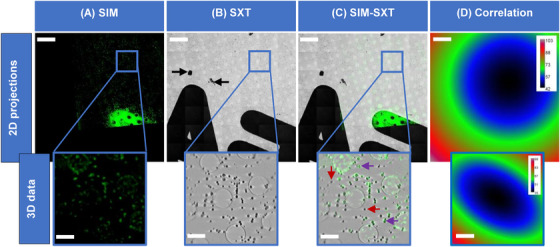
Qualitative correlative microscopy of AV‐ZIKA‐2 (batch 2) showing AV‐ZIKA‐2 in fluorescence (green particles) and X‐rays (grey objects). (A) Green fluorescence data obtained through cryoSIM reporting the immunolabelling of AV‐ZIKA‐2 on the grids. (B) X‐ray mosaic of same region in (A) obtained through cryoSXT. (C) Correlation of fluorescent and X‐ray data. (D) Heatmaps showing that the correlation error (TRE) for the representative 2D correlation in (C) ranged from 42 to 103 nm when 30 points were used for 2D correlation and 35–98 nm when 30 points were used for 3D correlation. These 2D and 3D TRE values depict effective correlation with minimal correlation error. The dark clumped particles (black arrows) in (B) are inert 250 nm gold nanoparticles added to the sample to provide high‐contrast features to aid data in processing as well as correlation. Blue boxes in (A)–(C) are insets highlighting 3D representative data. Purple arrows in (C) are pointing at AVs with fluorescence signals while the red arrows are pointing at AVs with no fluorescence signals. Scale bars: 2D projections (A)–(C) = 10 μm, 3D insets (A)–(C) = 2 μm

Following data collection and reconstruction of data from both microscopes, fluorescence (Figure 2) and X‐ray absorption data (Figure 3) were correlated using eC‐CLEM.^49^, ^50^, ^51^ Correlation of these datasets transcends observational purposes and allows the harvesting of quantitative information (Figure 4). X‐ray mosaics and tomograms reliably read out AV sizes given the achievable resolution of up to 25 nm,^33^ but on its own, SXT cannot unambiguously define intensity and antigen loading of AVs. This information is provided by the SIM data which provides pixel intensity profiles for the AVs in question based on their fluorescence. Hence, AVs intensity profiles from fluorescent SIM data (Figure 4A) and AVs sizes from X‐ray mosaics (Figure 4B) can be superimposed for the unambiguous assignment of both structural and chemical trends in AV subpopulations. For our purposes, correlation was done in eC‐CLEM using 32‐bit X‐ray data (Figure 4B). Image sizes were matched across SIM and SXT data and all slices along the *z*‐axis for 3D SIM were summed to produce a single 2D maximum intensity field of view (2D SIM, Figure 4A). Thirty common registration points were selected in both X‐ray mosaics and fluorescence images for increased correlation accuracy and minimal correlation error (tens of nanometres; Figure 4D). Aiming for high‐accuracy correlation is important because, these correlated 2D datasets served as source images during parameter assessments in Fiji. Registration points were distributed across the FOV to achieve uniform correlation accuracy in the entire FOV and prevent distortion or warping.^43^, ^50^ The 2D and 3D target registration errors (TRE) ranged from 42–103 nm and 35–98 nm (Figure 4D), respectively, depicting high‐fidelity correlation with minimal error.

The superimposed 2D data were saved as a merge of two images (Figure 4C). This merged 2D data contained a transformed fluorescent image now superposed with objects seen in the corresponding X‐ray data (Figure 4C).

### 5.2 Quantitative correlation of images in Fiji

**FIGURE 5:**
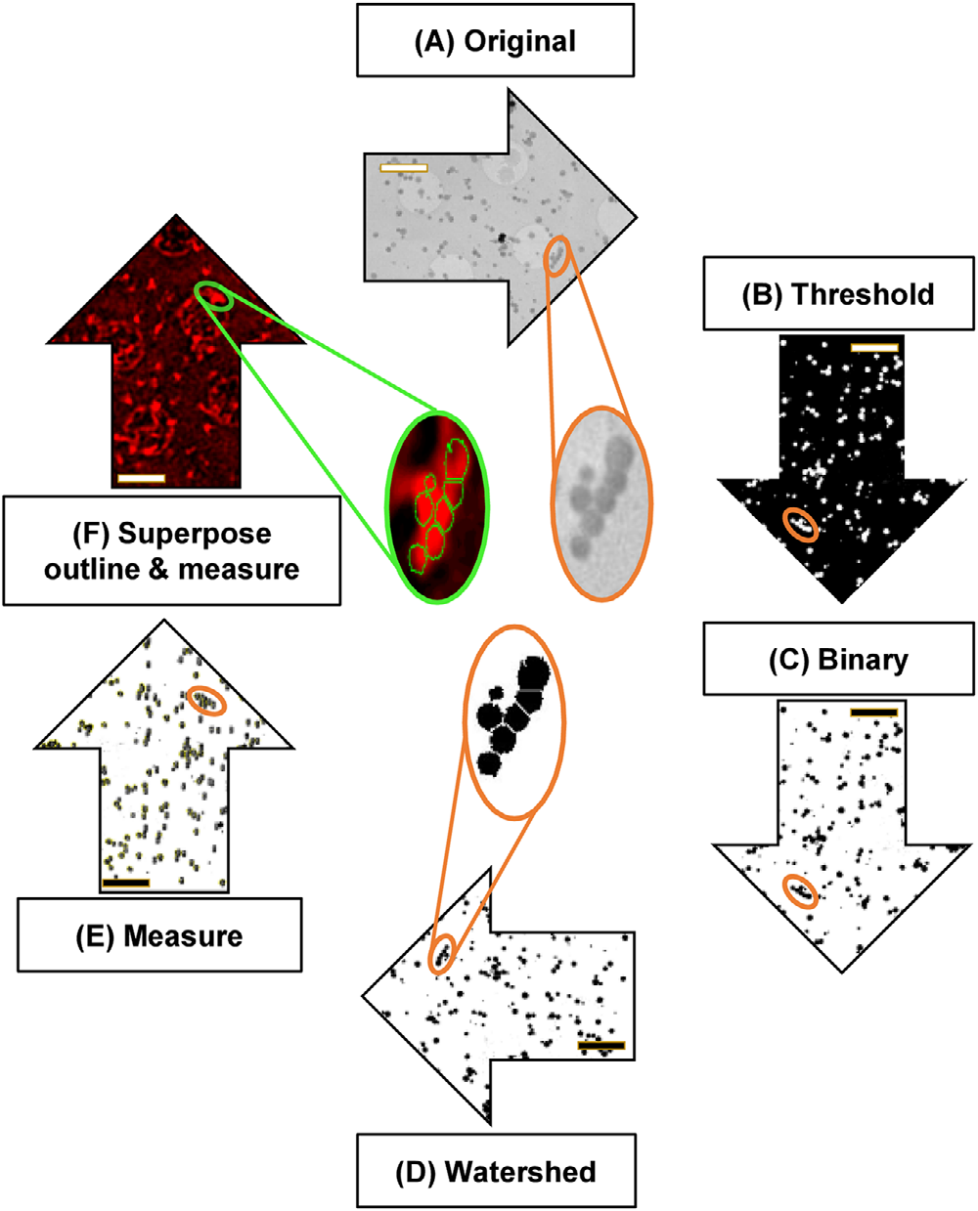
Manual image processing workflow for the quantitative correlation of X‐ray and SIM images showing AV‐ZIKA‐2. (A) Cropped out region from the original X‐ray mosaic. (B) X‐ray mosaic after thresholding. (C) Thresholded image was converted to 8‐bit binary. (D) Watershed filter was used to demarcate joined AVs. (E) Diameter of AVs were determined from X‐ray image. (F) Outlines of objects measured in (E) were superimposed on SIM data to measure fluorescence intensity. AV‐ZIKA‐2 fluorescence signal shown here in red. Scale bars = 2 μm

Using Fiji (ImageJ),^52^ suitable sub‐areas from 2D X‐ray mosaics (Figure 5A) were cropped and used to define the reciprocal areas in their 2D SIM correlates to preserve one‐to‐one identity. Pixel to size ratio were set depending on which data (SIM or SXT) was being analysed.

Data were then thresholded using the *isodata thresholding* method (Figure 5B), an iterative procedure which separates background from signals/objects by obtaining the mean of background versus object intensities.^53^ Thresholding is important as particle analysis can only take place on thresholded data which has all of its background noise subtracted. Thresholded images were further converted to binary (required for object size estimation; Figure 5C) and a watershed filter was applied to artificially separate adjoining AVs (Figure 5D). The resulting image was used to identify AV outlines (Figure 5E) according to the Feret's diameter (maximum distance between edge points). Objects were accepted provided they were (a) above 5000 nm^2^ in area, (b) displayed a circularity of 0.4–1.0 (1.0 denotes a perfect circle) and (c) did not reside on the edges of the selected 2D X‐ray area; all objects were then added to an ROI manager. ROIs retaining embedded metadata on *xy* coordinates and numbering order were saved as a zipped ROI folder. The corresponding 2D SIM cumulative fluorescence intensity data was then opened, and all pixels below 100 counts were set to a uniform background threshold of 100. Then, 100 counts were subtracted from all pixels in the dataset to assign a zero value to all spurious background noise signal. Pixel intensity was selected as a primary parameter and identified ROIs were superposed on it. If needed, identified object outlines were manually adjusted to correct for minor correlation discrepancies arising from correlation in eC‐CLEM. Integrated density measurements were then calculated for all AVs (Figure 5F).

Given the data analyses process here, different graphical representations of these quantitative correlated traits (fluorescence intensity and AV diameter reflecting antigen presence and vesicle size respectively) can be plotted for interpretation and microanalysis of the distribution, clustering, trend and variation in antigen loading of subpopulation of AVs.

### 5.3 Semi‐automated morphometric analysis of 2D AV X‐ray data

**FIGURE 6:**
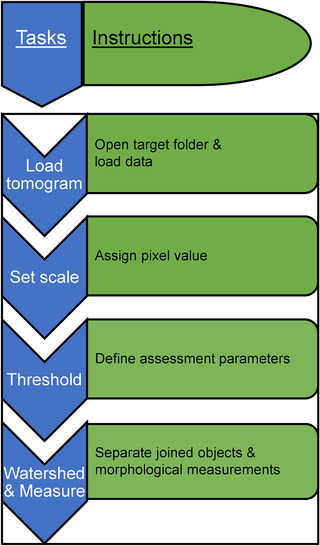
Chart showing the list of instructions carried out as contained in the MorphORBS macro‐script used in semi‐automated analysis of AV sizes from 2D X‐ray mosaics

Areas were cropped out of X‐ray mosaics and fed into a semi‐automated analyses pipeline we constructed based on custom‐written macroinstruction scripts (macros) in Fiji (MorphORBS) (Table 2). This macro‐run pipeline delivered high‐throughput characterisation of these AVs at beamline B24 and was configured to queue up all the settings previously described. It is important to note here that any potential data distortion because of filtering is countered by the number of AVs analysed. Using this approach (Figures 6 and S1), AV sizes were generated and transferred to a spreadsheet for collation and frequency distribution analyses

**TABLE 2:** Naming convention for custom‐written macro‐scripts

Name	Naming convention	Function
MorphORBS	Analysis of objects	**M** orphology **o** f **R** ound **B** iological object **S**
SegORBS	Segmentation of objects	**S** egmentation **o** f **R** ound **B** iological object **S**

### 5.4 Segmentation of AVs with 3D X‐ray data

**FIGURE 7:**
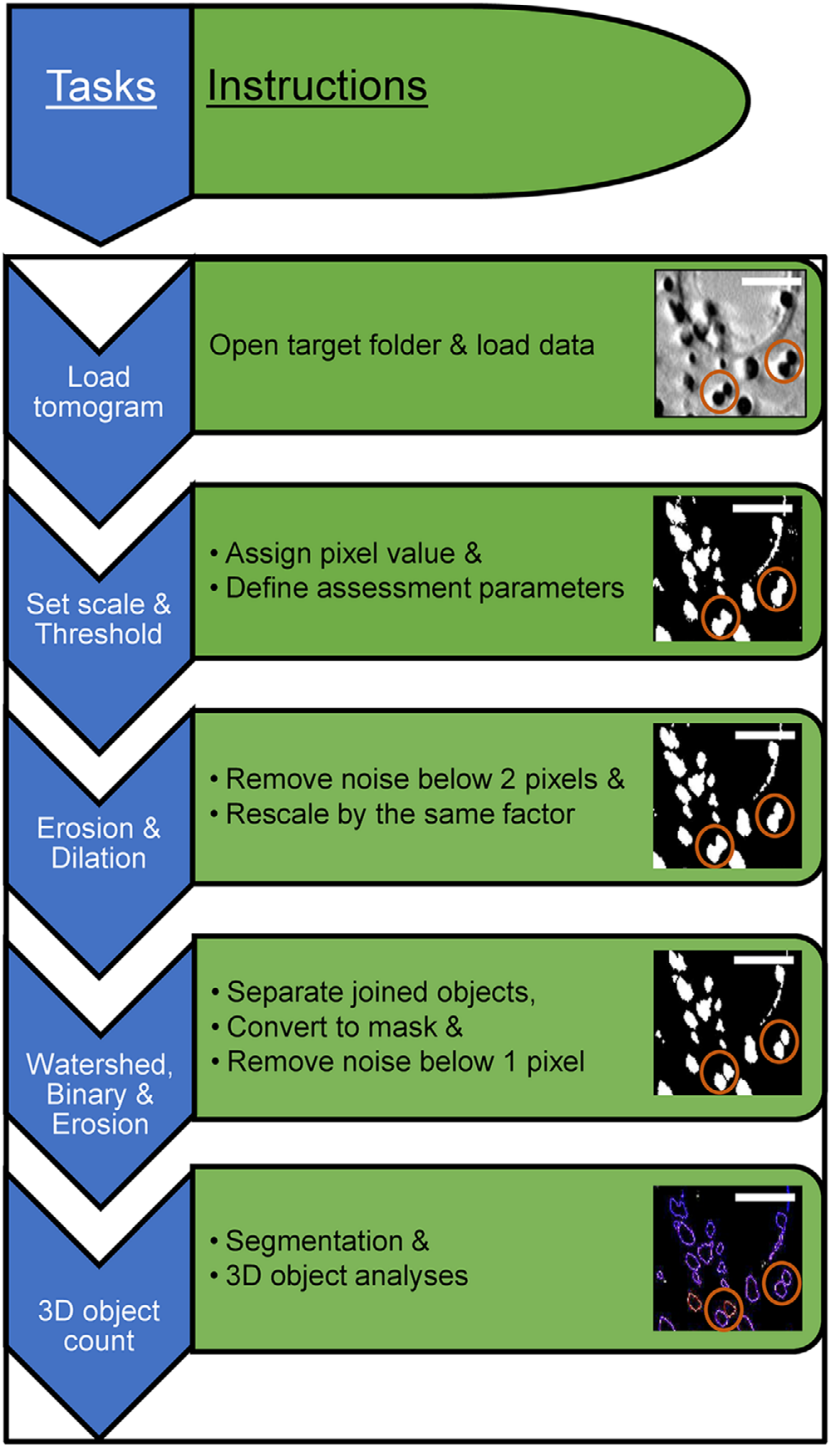
Chart showing the list of instructions carried out as contained in the SegORBS macro‐script used in semi‐automated segmentation of 3D AV datasets. Circles delineate filtering effects during the segmentation and analyses process. Scale bars = 1 μm

We constructed a separate Fiji‐based macro (SegORBS) (see Table 2) to segment and analyse 3D X‐ray tomograms. Here, reconstructed tomograms were trimmed to volumes containing clearly visible objects. The script was written to automatically adjust brightness and contrast, set the data to the desired scale, threshold the data and convert it to mask. Two‐pixel erosion and dilation filters were applied to remove extraneous features (such as sample carrier surfaces) proximal to AV edges. To accurately assign boundaries between AVs, the script incorporated a 3D watershed filter which separated objects by a factor of 1 pixel and converted the processed data to 8‐bit binary. Persistent background or artefacts were removed with an additional step of 1‐pixel erosion. The output was then used to identify AVs and generate surface areas and volumes after 3D object analyses. The latter also output object or surface maps for all segmented AVs (Figure 7). Surface areas were further used to calculate AV diameter.

### 5.5 Statistical analysis

Descriptive statistics which were used in this study were: (1) frequency distribution, (2) measures of central tendency: mean, mode and median and (3) measures of variability: range and standard deviation (SD). In addition, mean nearest neighbour distance (NND) and nearest neighbour index (NNI) were estimated. Statistical inferential analysis was performed with two‐tailed unpaired *t*‐test. Significance level (*α*) was set at 0.05, where *p* ≤ 0.05 was considered to be statistically significant.

## 6 RESULTS AND DISCUSSION

### 6.1 Traits in AV populations

Multiple samples were prepared for different AV batches for each pathogen (see Table 1) and multiple regions within each sample were imaged. The power of our correlative cryo‐imaging approach and workflow lies in the promise of both ultrastructural characterisation of AV vesicles in a sample and the disambiguation of fluorescently targeted chemical information (antigen loading) on the surface of these same vesicle population.

**FIGURE 8:**
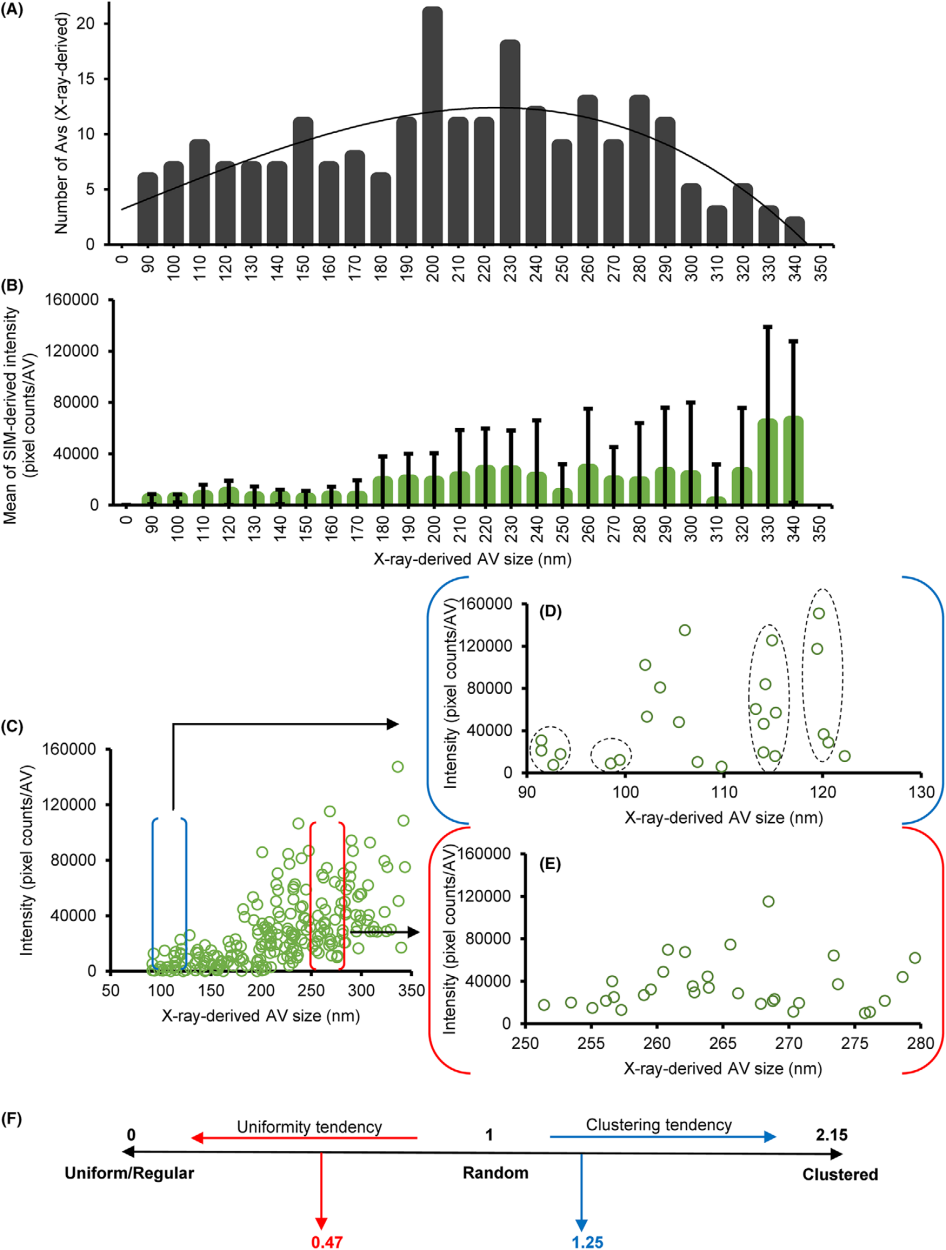
Quantitative correlative microscopy of AV‐ZIKA‐2, showing AV‐ZIKA‐2 X‐ray data in black bars and fluorescence intensity data in green bars and circles. (A) Graph showing frequency distribution of AV‐ZIKA‐2 sizes in increments of 10 nm. These diameter results were obtained from X‐ray mosaics. (B) Graph showing frequency distribution of AV‐ZIKA‐2 pixel mean intensities within defined AV size bands in increments of 10 nm. Data is shown as mean ± SD. (C) Graph showing frequency distribution of discrete AVs pixel intensities within defined AV size bands in increments of 50 nm. A blown out graphical representation of the frequency distribution of discrete AVs pixel intensities in (C) within defined AV size bands of (D), 90–120 nm. (E) 250–280 nm. (F) NNI Scale showing distribution zones and tendencies. Black dotted circles in (D) represent cluster definition

Variability in the size of vesicles (diameters from a few tens to a few hundred of nanometres) within AV populations was immediately noticeable in SXT data. Moreover, correlated data indicated that within any batch of AVs there are brightly fluorescent vesicles (indicating viral antigens are present) and others with limited or no fluorescence (indicating the absence of a viral load) (Figure 4C). To further explore these observations, we focused on AV‐ZIKA‐2 (Figure 4) and manually collated AV incidence within increments of defined diameters (each class +10 nm progressively). These were plotted against the total number of AVs contained within each class (Figure 8A) and analysed with respect to skewness which measures the symmetry of a distribution and kurtosis which measures whether the frequency distribution is ‘tailing’ as well as its ‘peakedness’. ^54^, ^55^ The AV‐ZIKA‐2 vesicle population had a skew of 0.22 and kurtosis of −0.077 which indicate a normal distribution of sizes. The modal AV size was at 200 nm, with 230 nm sized AV‐ZIKA‐2 coming in as the second highest occurring size. Given that these vesicles are harvested from a mammalian ‘production’ cell line (Vero cells; African green monkey kidney epithelial cells), the size distribution we observe is likely to reflect vesicle tropism that is representative of the maternal cell line and its capacity to package and expel exosomes. The consideration here would be relevant to AV production if it was to be achieved in another cell line which could likely output AV populations with different distribution of sizes. In the event that either the primary immune response or the subsequent uptake by host cells are dependent on vesicle size, knowledge of these values would be absolutely necessary for assessment of vaccine potency (Table 3).

**TABLE 3:** Template for quality assurance using cryoCLXM

**Batch parameters**	**Values required**	**Possible implications**
Population density	Number per area (#/μm^2^)	‐Reproducibility, ‐Production standardisation
	# of objects in a size range per area (#/μm^2^)	
Vesicle structure	Size range	‐Production specificity ‐Product purity
	Mean/modal size	
	Skew/kurtosis of size distribution	
Antigen loading	Mean pixel intensity	‐Variability in protein decoration
	Standard deviation of pixel intensity	
Size clustering	Nearest neighbour distance Nearest neighbour index	‐Cell‐based restrictions on product architecture

Using the same vesicle distribution, corresponding cryoSIM data was analysed (Figure 8B). Antigen presentation (indirectly measured here through fluorescence intensity) and variability was lower in smaller‐sized AVs (90–170 nm) but higher in larger AVs (180–340 nm). This suggests that AV vesicles carried a variable load of antigens in larger AVs when compared to smaller AVs (*p* < 0.05). For instance, the mean (± SD) fluorescence intensity of AVs in class widths of 100–110 nm and 200–210 nm were 3693 ± 5583 and 22,287 ± 18,166 counts per AV respectively (*p* = 0.00043, where mean cumulative single AV intensity in this sample was 18,491 counts). This implies that antigen loading is dependent on total vesicle surface with smaller vesicles displaying a more ‘saturated’ outer membrane leaf. The implication here is that AV formation in our ‘production’ cell line is likely dependent on localised incidence of viral antigen rafts or preferential recruitment of these antigens at budding sites.

Further analysis was performed at specific size ranges of the AV populations to ascertain the behaviour of subpopulations with respect to vesicle size. Microanalyses of neighbour‐to‐neighbour distance showed a clustering propensity in smaller vesicle subpopulations while larger vesicles distribute more evenly across sizes (Figure 8C–E). Nearest neighbour distance (NND) analysis^56^ for distances between marked clusters gave a mean NND ± SD between 7 clusters of 3.1 ± 1.2 nm and 1.2 ± 0.5 nm for AV subpopulations ranging from 90 to 120 nm (Set 1) and 250 to 280 nm (Set 2), respectively. Two‐tailed unpaired *t*‐test was used to analyse the mean NND of these subpopulations of AV‐ZIKA‐2 and a significant difference (*p* = 0.004) was observed. Furthermore, the nearest neighbour index (NNI), which ranges from 0 to 2.15 (with 0 in this case denoting uniform distribution, 1 denoting random distribution and 2.15 denoting aggregation/clustering of sizes) was calculated. The NNI range scale interpretation was modified here and adapted to define our data appropriately because, distribution was not interpreted merely as distances between individual AV sizes, but as distances between marked clusters of AV sizes. Hence a larger NNI would denote aggregation of subsets of AVs since aggregation would lead to segregation and increased distance, while a smaller NNI would denote a spread of AVs thereby leading to reduced distance. The formular below was used to estimate NNI:^56^

$$NNI=\frac{2\bar{d}\left(n-1\right)}{L},$$

where $\bar{d}$ = mean NND, *L* = Length sampled; in this case, it is the size range = 30 nm, *n* = number of points (in this case, number of gaps between clusters).

The nearest neighbour index (NNI) of Sets 1 and 2 were 1.25 and 0.47, respectively, further supporting these findings: clustering tendency for smaller vesicles (Set 1) and uniformity tendency for larger vesicles (Figure 8F). These distinct structural traits in AV subpopulations are potential indicators of mechanistic dependencies in AV production overall. As such they could provide valuable insight into the exosome production machinery of cells and indicate that there is an innate architectural geometric restriction that predefines the packaging sizes of only the small AVs during their budding from their host cell.^57^ During vaccines development, this salient information on assumptive exosomes size restrictions can be applied during centrifugation to optimise AVs yield in specific size bands.

### 6.2 AV batch‐to‐batch variability

**FIGURE 9:**
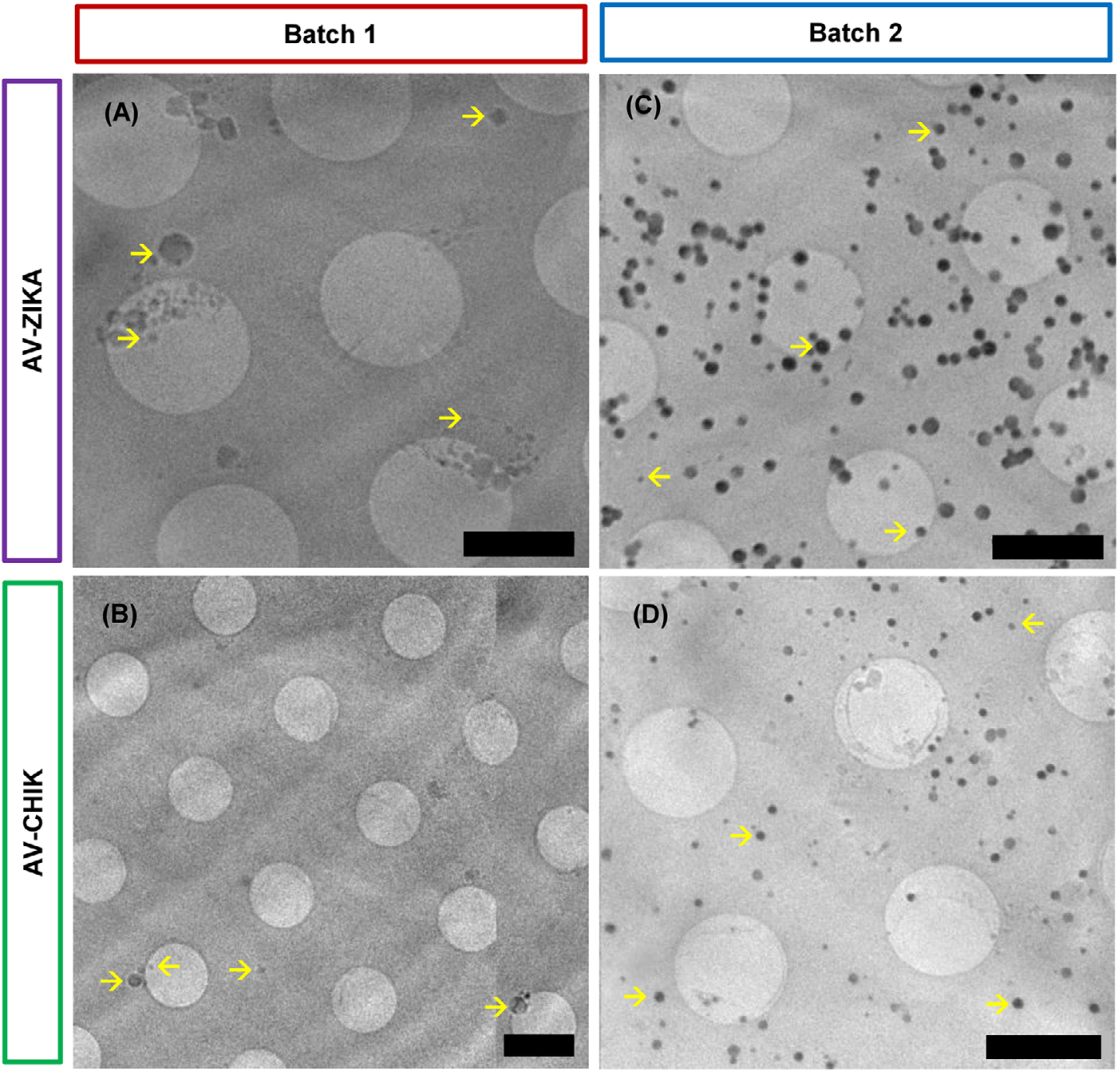
Representative slices of 3D X‐ray tomograms of different batches of AVs showing variabilities in manufactured batches. (A) Tomogram slice of AV‐ZIKA‐1 captured with a 25 nm zone plate, with size range from 125 to 600 nm. (B) Tomogram slice of AV‐CHIK‐1 captured with a 40 nm zone plate with size range from 75 to 400 nm. (C) Tomogram slice showing AV‐ZIKA‐2 captured with a 25 nm zone plate, sizes ranged from 88 to 300 nm. (D) Tomogram slice showing AV‐CHIK‐2 captured with 25 nm zone plate with sizes ranging from 100–200 nm. Yellow arrows indicate variably sized AVs. Scale bars = 2 μm

The methods and protocols described in this study were applied to different batches of Zika and Chikungunya AVs to ascertain the reproducibility of the manufacturing process with respect to size, density and antigen expression (see Table 1). Given that samples were layered on a flat surface for imaging, density was calculated as number of vesicles per μm^2^ of the grid. AVs for both pathogens from one production batch were sparsely distributed (0.06 ± 0.03 and 0.08 ± 0.03 μm^−2^ for AV‐ZIKA and AV‐CHIK, respectively) and variable in features compared to the well‐defined spherical geometry populous samples from the second batch (1.24 ± 0.51 and 1.40 ± 0.34 μm^−2^ for AV‐ZIKA and AV‐CHIK, respectively; Figure 9). Population densities were statistically analysed using unpaired *t*‐test and this was shown to be significantly different (*p* < 0.05) among batches. Samples for imaging were prepared concurrently following an identical protocol so the observed discrepancies were likely a reflection of production variability at Seagull BioSolutions Private Ltd. Given the sparsity of material in batch 1 formulations, only manual measurements of AV sizes were performed (Figure 9). Cross‐batch variability further emphasises the need for imaging and quantification of AV samples before these are used for testing or treatment.

### 6.3 Characterisation of AV subpopulations using MorphORBS

**FIGURE 10:**
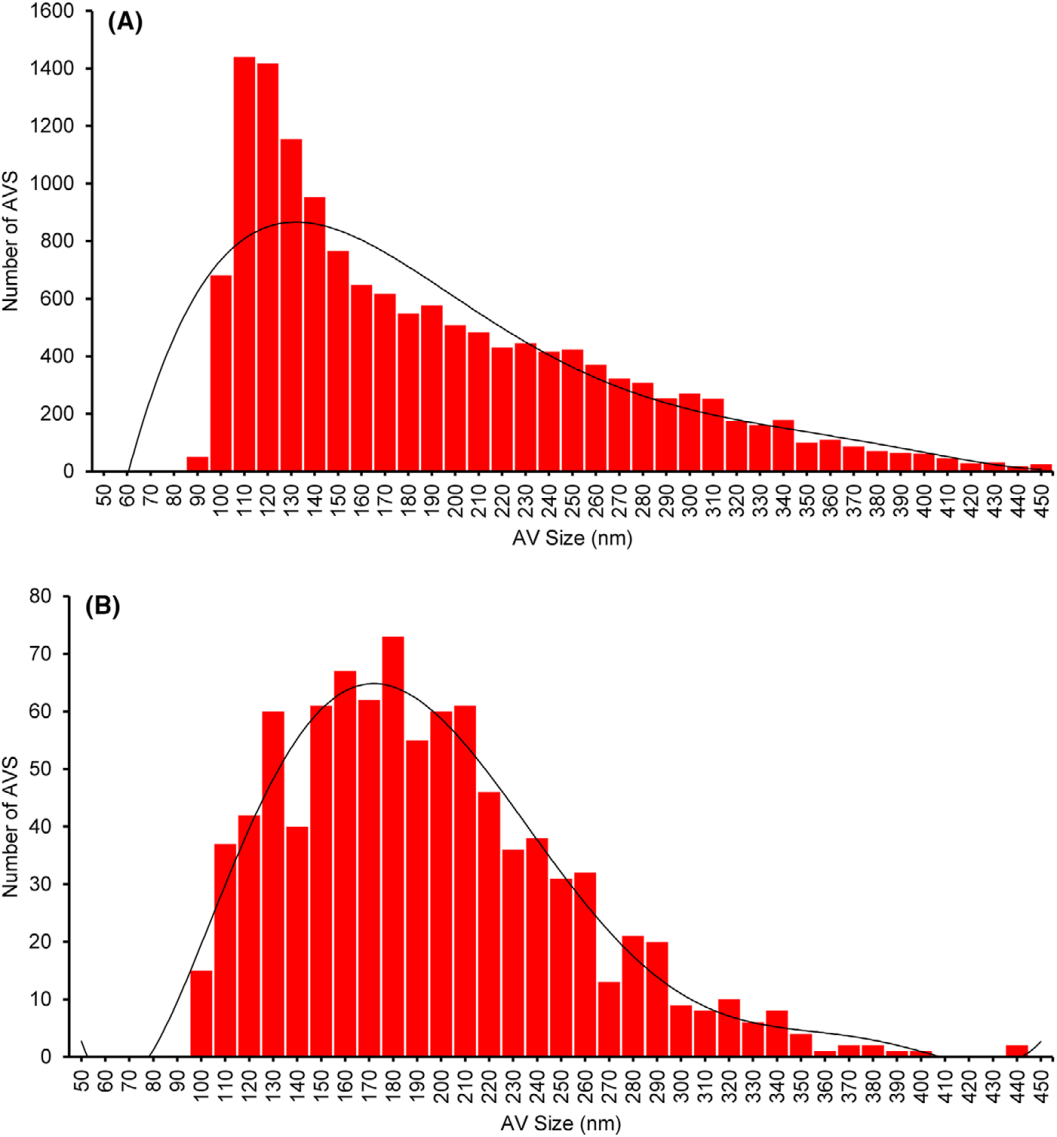
Size distribution of AV‐ZIKA‐2 and AV‐CHIK‐2 determined by semi‐automatic analysis of X‐ray mosaics using MorphORBS. (A) Average diameter of AV‐ZIKA‐2 and (B) average diameter of AV‐CHIK‐2

Average diameter for AVs was computed as a mean of the minimum and maximum Feret's diameter (a standard measurement option in Fiji; Figure 10). The distribution of vesicle diameter in AV‐ZIKA‐2 (skewness = 1.44; kurtosis = 1.89) and in AV‐CHIK‐2 (skewness = 0.70; kurtosis = −1.02) when plotted assume a Gaussian distribution. The modal average size for AV‐ZIKA‐2 is 110 nm with object sizes ranging from 50 nm to 450 nm while the modal average size for AV‐CHIK‐2 is 180 nm with vesicle sizes ranging from 100 to 450 nm. AV‐ZIKA‐2 samples have a narrower range of AV sizes with a smaller modal as compared to AV‐CHIK‐2 (Figure 10) which is suggestive of host cell packaging bias depending on the antigen expressed.

Automation of the process though MorphORBS allows the evaluation of a robust number of AVs (ranging from hundreds to thousands) in a rapid fashion compared to any manual measurements.

By assessing AV diameters using 2D X‐ray mosaics and assuming a perfectly spherical geometry for all AVs, radii and then volumes of 3D spheres could be extrapolated allowing the fast and reliable estimation of volumetric information from 2D data where needed. To directly measure volumes in AV samples 3D X‐ray tomograms can be used.

### 6.4 Segmentation and analysis of AV 3D data using SegORBS

**FIGURE 11:**
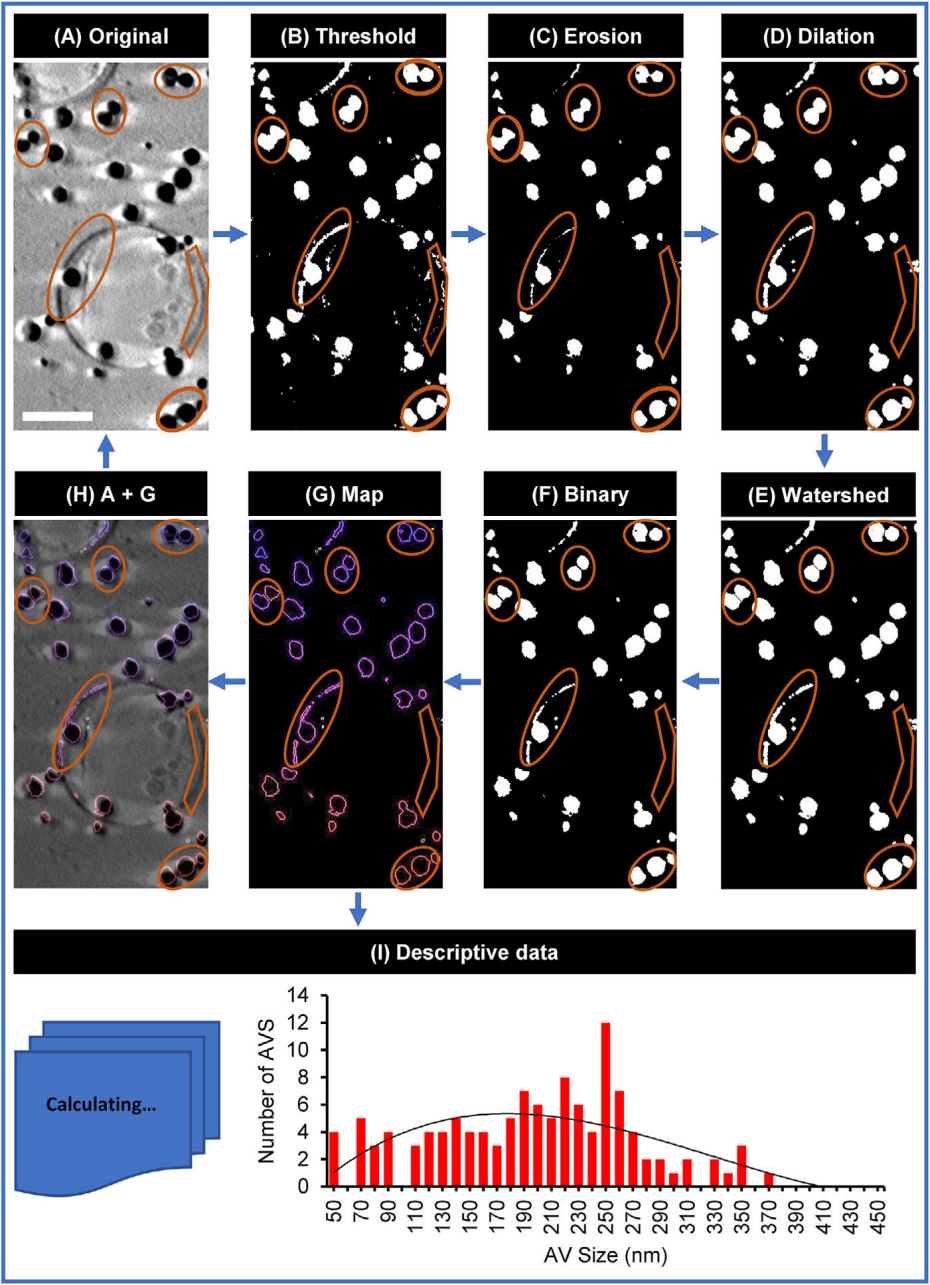
Semi‐automated segmentation of 3D tomograms of AV‐ZIKA‐2 using SegORBS macro‐script. The workflow starts from (A) trimming the 3D tomogram to a smaller volume to cut off blank areas in *Z*. This facilitates faster processing as well as concentrates on the relevant datasets. (B) Thresholding. (C) Erosion filter subtracts TEM grid background. (D) Dilating back pixels by the same erosion factor (e.g. ×2). The logic behind this is that if erosion removed background artefacts to zero, then by dilating it again by the same factor of e.g. ×2 used in erosion, those eroded artefacts at zero will remain zero and cleaned off, whereas objects which were not artefacts but genuine will be dilated back to their original state (e.g. 0 × 2 = 0 and 2 × 2 = 4). (E) Application of a thin watershed filter to demarcate joined or clumped AVs. (F) Conversion of tomogram to binary. (G) Surface map generated during 3D object morphometric analysis. (H) The surface map was merged with the tomogram manually. (I) Graphical representation of size distribution. Orange shapes in (A)–(H) are highlighting segmentation results obtained from each filter applied. Scale bar = 1 μm

AV tomograms were segmented semi‐automatically by the custom‐written macro‐SegORBS (see Table 2 and Figure 7). The prescribed application of selected image filters (Figures 7 and S2) for automated 3D counting^58^ and analyses of AV data can provide segmented volumes as well as population measurements (volume, surface area, voxels per object volume, voxels per vesicle surface) providing a detailed view of AV microstructure in a given AV sample (Figure 11). The SegORBS pipeline is adaptable and each stage of processing should be tested independently (application of respective filtering step in Fiji manually) to ensure that parameters are chosen to suit the sample at hand. However, strings of filters selected for segmentation and 3D volumetric analysis should be kept consistent throughout the analyses process across samples to ensure reproducibility and uniformity in the selection of objects. In addition, it is necessary to sample enough objects (at least several hundreds of AVs per sample) to avoid statistical bias.

Similar trends are observed with both MorphORBS‐ and SegORBS‐derived AV sizes in the form of the normal distribution patterns and a modal value derived from both methods to be 150 nm (Figures 10A and 11I). The skew of the distributions produced using the two methods varies slightly with different extremes on the larger vesicle population (370 nm with SegORBS as opposed to 450 nm with MorphORBS). Given that the 3D analysis is entirely observational by nature while 2D analyses extrapolate the third dimension through an assumption of perfect spherical geometry, it is likely that the overestimation of maximum AV size is representative of an inherent bias of the 2D analyses towards overestimation of AV volume. However, great care needs to be taken when using SXT data. The current SXT implementation restricts the achievable angles of rotation (−70° to +70°) during data collection (projections are recorded from the same sample at different angles to generate a 3D view) thereby leading to anisotropic resolution across dimensions and the apparent elongation of objects along the beam direction (*z* axis).^59^ Hence, when deciding to use 3D quantitative data from SegORBS segmentation, it is important to apply a single correction factor across board to correct for this method artefact and minimise volume expansion.^60^, ^61^

Both types of processing (MorphORBS and SegORBS) proved well suited to microanalyses of AV samples (across antigens and batches) and data are consistent within each processing regime. The 3D analyses appear analytically more robust, however, 2D analyses allows markedly larger sampling at a fraction of the time. Therefore, analyses of 3D data using SegORBS could be used to calibrate parameters in MorphORBS before using the later for semi‐automated bulk processing of AV data across batches in an expedient and accurate fashion.

### 6.5 Assessment of cell viability post exposure to AVs in mammalian cell populations

**FIGURE 12:**
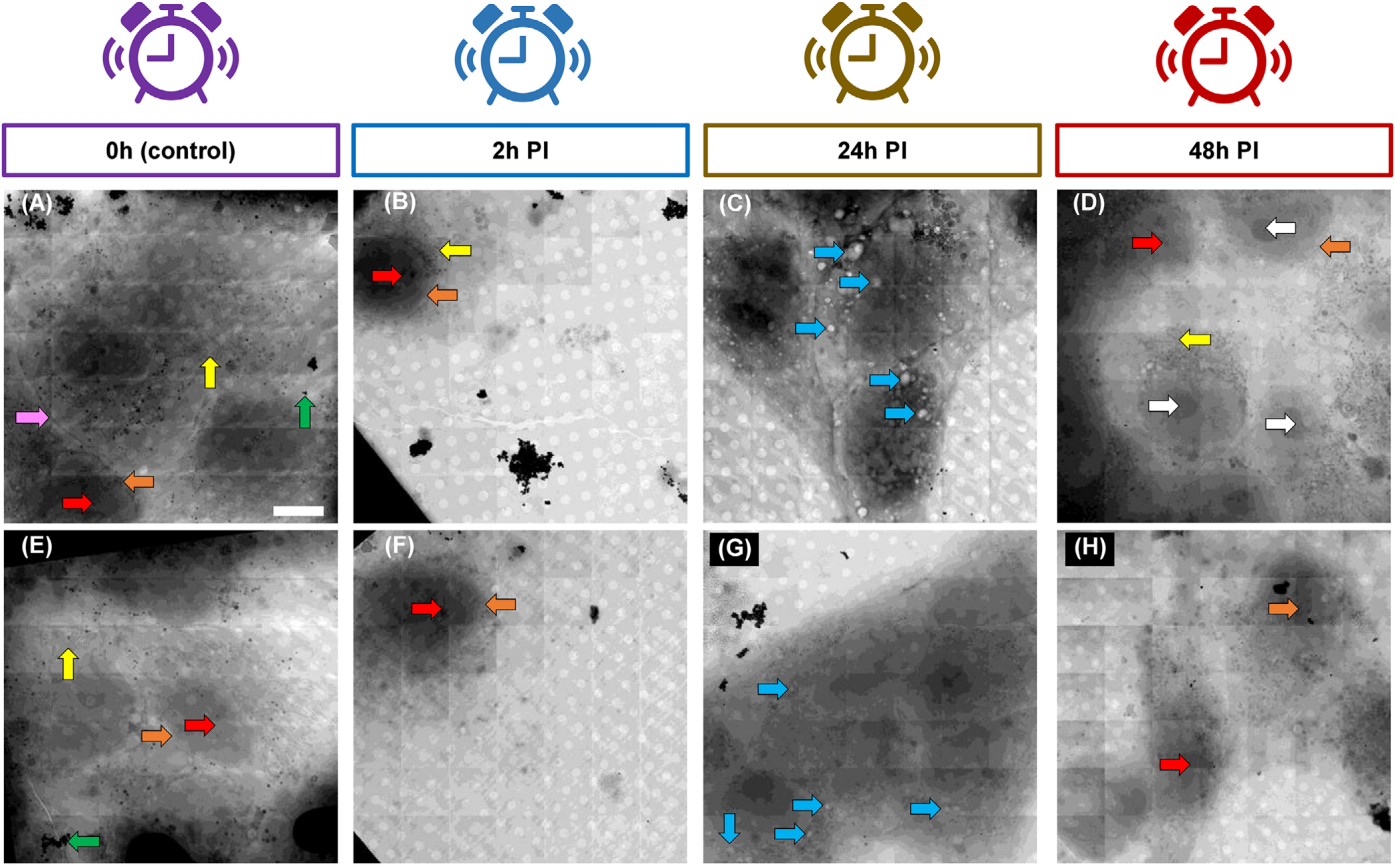
Representative 7 × 7 2D X‐ray mosaics of Vero cells at different time points of infection with AV‐ZIKA‐2 and AV‐CHIK‐2. (A) Mosaic of uninfected control cells showing normal gross morphology of cellular organisation. (B) Mosaic showing an AV‐ZIKA‐2‐infected cell at *T* = 2 h. (C) AV‐ZIKA‐2‐infected cells at *T* = 24 h. (D) AV‐ZIKA‐2‐infected cells at *T* = 48 h showing full recovery to normalcy. (E) Mosaic of uninfected control cells showing normal gross morphology of cellular organisation. (F) Mosaic showing an AV‐CHIK‐2‐infected cell at *T* = 2 h. (G) AV‐CHIK‐2‐infected cells at *T* = 24 h. (H) AV‐CHIK‐2‐infected cells at *T* = 48 h showing clear recovery. White arrows (nucleolus), red arrows (nucleus), orange arrows (nuclear envelope), yellow arrows (mitochondria), green arrows (250 nm gold nanoparticles as fiducial), blue arrow (vacuoles) and purple arrows (cell membrane). Scale bar = 10 μm

To test the effect of AVs in an unexposed population of cells in culture we used Vero cells and exposed them to different AV batches. We then captured the cellular ultrastructure at different time intervals post‐exposure by sample vitrification and tested for possible antigen expression on the cell surface (see Section 2) (Figure 12).

Host cells appeared healthy before AV exposure with clear nuclear regions, well defined cytoplasmic organelles and continuous plasma membrane (Figure 12A, E). By 2 h, post‐exposure (PE) cells remained viable though visibly contracted (Figure 12B, F). At 24 h, PE cells showed clear signs of stress (multiple cytoplasmic vacuoles) (Figure 12C, G), however, by 48 h PE, they appear recovered and exhibit normal cellular physiology (Figure 12D, H). At this time point, nuclei appear intact with clearly visible nucleoli, whereas in the cytoplasm, organelles such as mitochondria appear complete and elongated as seen in the control cells (cells could be seen actively dividing at 48 h PE (data not shown here). This type of *in cellulo* analyses could become an important assessment step in vaccine development, whereupon novel formulations are tested in native single cell populations as a proxy for studies on host responses in tissue and organisms during early R&D stages. It should however be stated here that even though our microscopy platform can generate high‐throughput datasets of AVs, given their field of view, they are limited in the number of full cell imaging they can output and are unlikely to deliver exhaustive whole cell population health statistics following exposure to vaccines.

### 6.6 ‘Second‐wave’ AV antigen expressions at host cell surfaces and the promise of prolonged immunity

**FIGURE 13:**
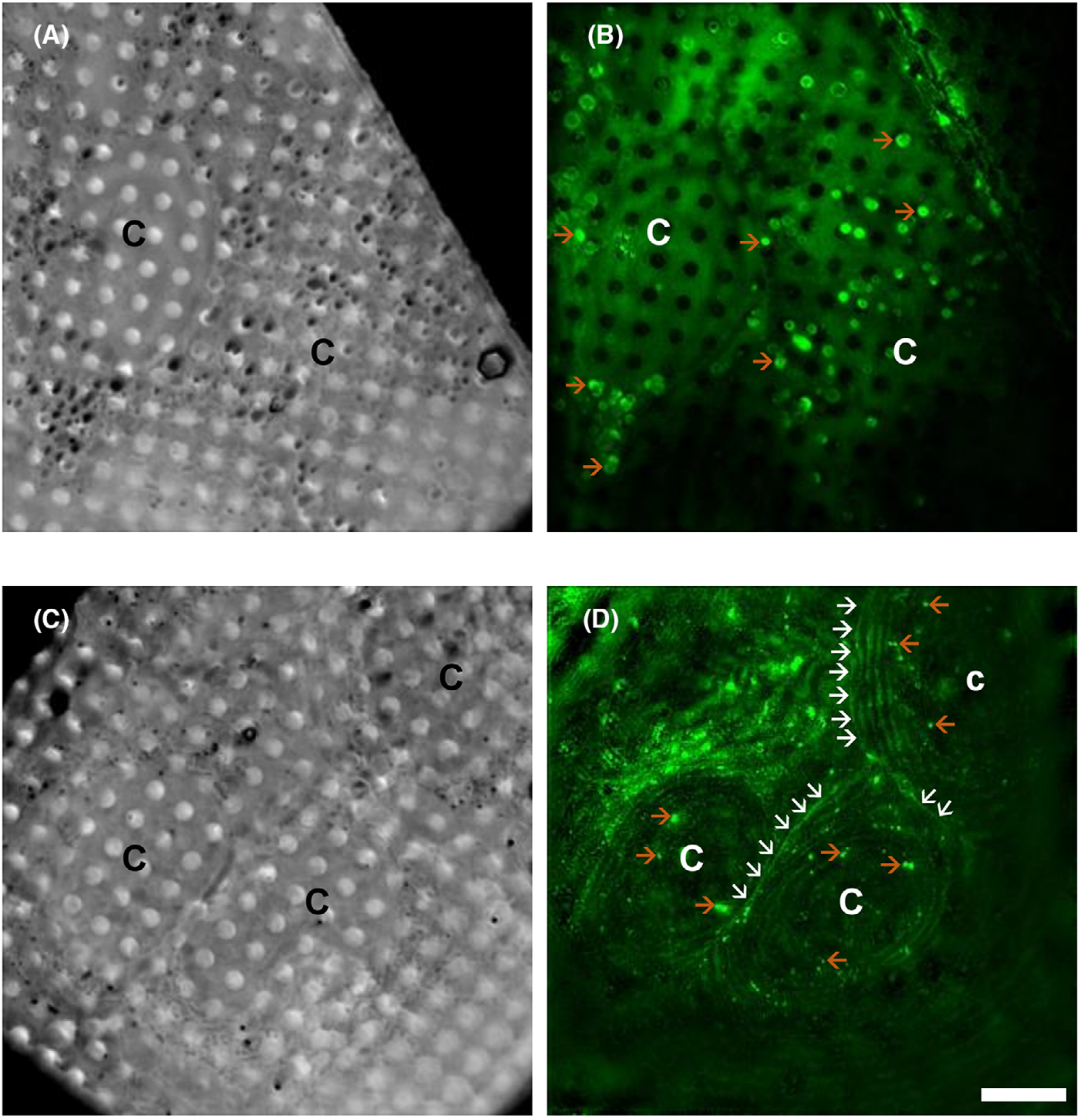
Time course of Vero cells exposed to AV‐CHIK‐2 to validate the second wave of antigen expression on cell surfaces and highlight the power of our cryoSIM platform. (A) Minimum intensity projection of 3D stacks of bright field data showing Vero cells at 1 h PI with AV‐CHIK‐2. (B) Maximum fluorescent intensity projection of 3D structured illumination data showing Vero cells at 1 h PI with AV‐CHIK‐2. (C) Minimum intensity projection of 3D stacks of bright field data showing Vero cells at 4 days PI with AV‐CHIK‐2. (D) Maximum fluorescent intensity projection of 3D structured illumination data showing Vero cells at 4 days PI with AV‐CHIK‐2. The white arrows are pointing at the fluorescently labelled antigen expression on the cell surface following translation from AV‐CHIK‐2 genome. The green fluorescence at the surface (white arrows) represents immunolabelled AV‐CHIK‐2, while the green fluorescence within the cells (orange arrows) represents endogenous autofluorescence. Different cells are denoted with C. Scale bar = 10 μm

Active virosomes confer to host cells the ability to express viral antigens which can then be displayed on the cell surface, thereby triggering a second ‘dose’ of antigen exposure for the immune system therefore prolonging the immune response. To confirm this mode of action in our samples, Vero cells were immune‐fluorescently labelled at different time points PE (1 h to assess immediate response and cytotoxicity and 4 days to assess viral protein expression) (Figure 13). No fluorescence signal was observed at the cell surface 1 h PE. Immunostaining was performed in culture and cells were neither fixed nor permeabilised. So, any fluorescence recorded inside the cells is due to native autofluorescent aggregates while fluorescence noted at the membrane edge of cells can only be due to the presence of AV antigens exposed on the outside face of the cell membrane and therefore accessible to the fluorescent antibodies that recognise them. We noted that antigen‐specific fluorescence is evident at cell edges and more distinct near the boundaries between neighbouring cells; it is expected that the cell boundaries are thicker and bulging because of mechanical pressure generated from contact with adjoining cells and therefore antigens present there would be partially stacked, giving a stronger fluorescence signal than other cell edges. This is clear confirmation of successful AV genetic load translation and translocation to the cell surface. It is likely that our results could provide a quantitative measure for AV efficacy although uniform expression of viral antigens across cells and cell surfaces is unlikely because it would be dependent on the cell type, variable rates of internalisation of AVs based on their composition and size as well as intracellular processing. Dosage should also be calibrated carefully to balance toxicity upon exposure with survival and optimal expression of viral antigens.

## 7 CONCLUSIONS

This work demonstrates clearly that a correlative cryo‐microscopy approach using both high‐resolution fluorescence microscopy and soft‐X‐ray tomography at cryogenic temperatures allows the characterisation of active virosome vaccine candidates and could efficiently complement protocols currently in place for similar vaccine formulations. Characterisation of AVs using our workflow highlights its feasibility at the R&D stage prior to scale‐up manufacturing. In this work, imaging data were collected for two AV vaccine batches and for each batch, multiple sample areas were investigated. The results were comparable across all similar sample batches showing the consistency and reproducibility of the vaccine technology and imaging techniques used. It was also suitable for two different AV vaccine types, showing that this approach is a versatile probing tool for the analysis of different manufactured batch types. This high‐throughput semi‐automated correlative vaccine characterisation pipeline at beamline B24 portrays a protocol for translational application during vaccine development to map out quality criteria within the context of medical regulatory submissions (pre‐clinical proof of concept) for AV products and other vaccine candidates. From the AV scale‐up manufacturing end, future works should focus on optimising scale‐up manufacturing and standardisation processes for AVs.

Biologically, this study demonstrates the potential of our integrated approach during early pre‐clinical trials to substantiate the survivability and recovery of cells following AVs incorporation, akin to vaccination in animals or humans. This is important because it is necessary to demonstrate that vaccine candidates do not destroy host cells; rather, that they are effective enough to induce immune responses without obliterating cell populations. In addition, remodelling activities and changes in cellular ultrastructure following AVs infection can be assessed using our imaging techniques. Seeing that these AVs were able to deliver a second wave of immune response as portrayed by the viral antigens displayed at the host's cell surface at 4‐day PE, we show that these active virosomes do deliver their design promise of prolonged immunity when compared to the traditional virosome‐based vaccine technologies.

Taken together, the morphometric characterisation of these AV particles and their interaction with mammalian cells show that our platforms and pipeline satisfy a need for high‐resolution imaging at the production level and can complement existing techniques in the vaccine development terrain by characterising vaccine candidates biophysically, in timed‐snapshots, multi‐colour (if necessary) and in 3D.

## CONFLICT OF INTEREST

The authors declare that there is no conflict of interest regarding the publication of this article.

## Supporting information

FIGURE S1. Chart showing the list of instructions and the desired tasks to be carried out as contained in the MorphORBS macro‐script used in automated analysis of AV size from 2D X‐ray mosaic. The lowest level of watershed filter (level 1) was selected for proper demarcation in the event of the occurrence of joined AVs in any FOV being analysed. The impact of this watershed is negligible and is cancelled by the large number of AVs analysed and the fact that size distribution is the desired parameter and not mean size of AVs

FIGURE S2. Chart showing the list of instructions and the desired tasks to be carried out as contained in the SegORBS macro‐script used in automated segmentation of 3D AV datasets. The lowest level of watershed filter (level 1) was selected for proper demarcation in the event of the occurrence of joined AVs in any FOV being analysed
