# Supplementary figures and images for: Correlative imaging using super‐resolution fluorescence microscopy and soft X‐ray tomography at cryogenic temperatures provides a new way to assess virosome solutions for vaccine development
Source: J Microsc. 2021 Sep 3;284(3):214–32. doi: 10.1111/jmi.13054 (PMC9292697; doi:10.1111/jmi.13054)

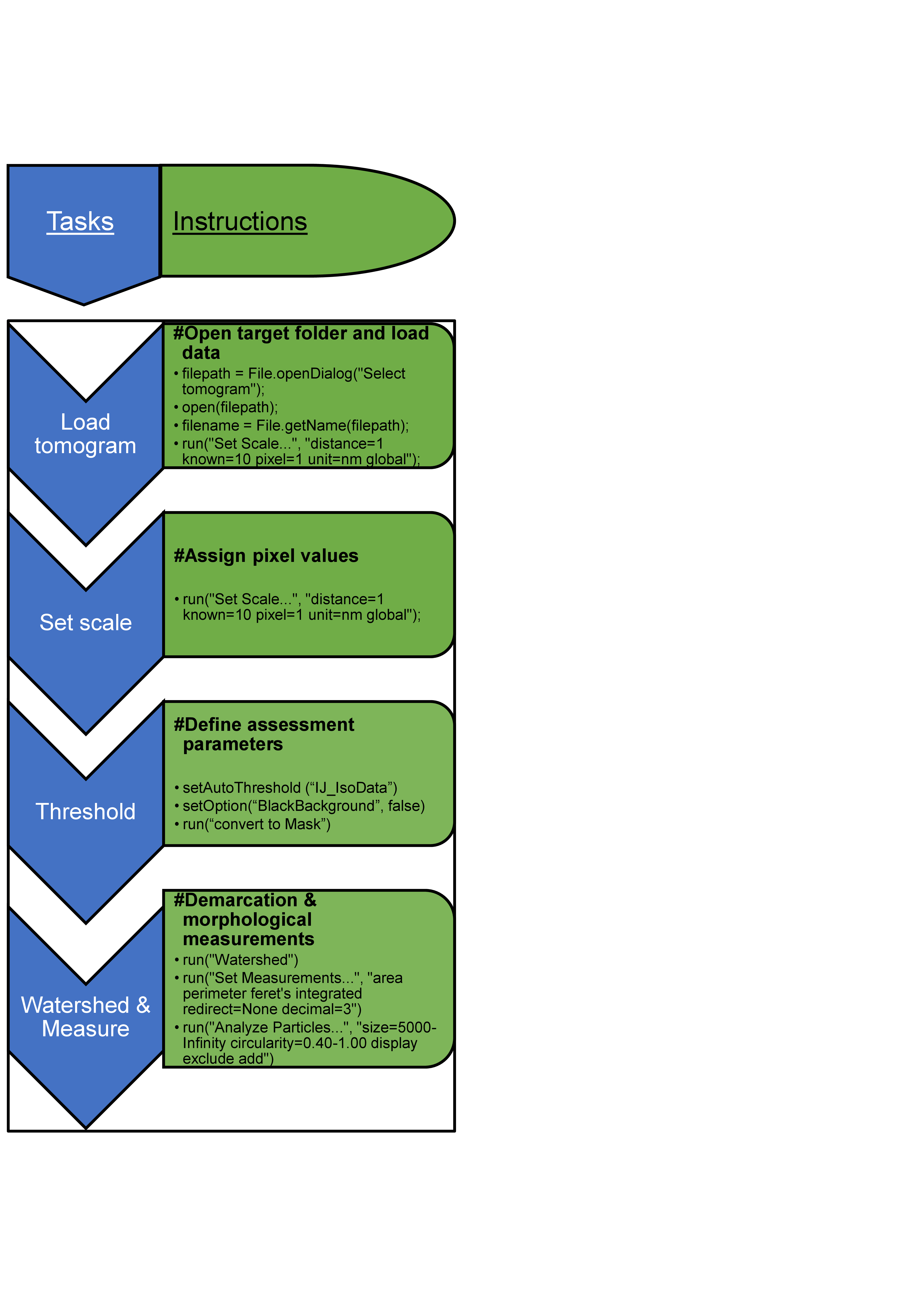

Supplement: Supplementary file 1 — FIGURE S1. Chart showing the list of instructions and the desired tasks to be carried out as contained in the MorphORBS macro‐script used in automated analysis of AV size from 2D X‐ray mosaic. The lowest level of watershed filter (level 1) was selected for proper demarcation in the event of the occurrence of joined AVs in any FOV being analysed. The impact of this watershed is negligible and is cancelled by the large number of AVs analysed and the fact that size distribution is the desired parameter and not mean size of AVs [file JMI-284-214-s001.tiff]

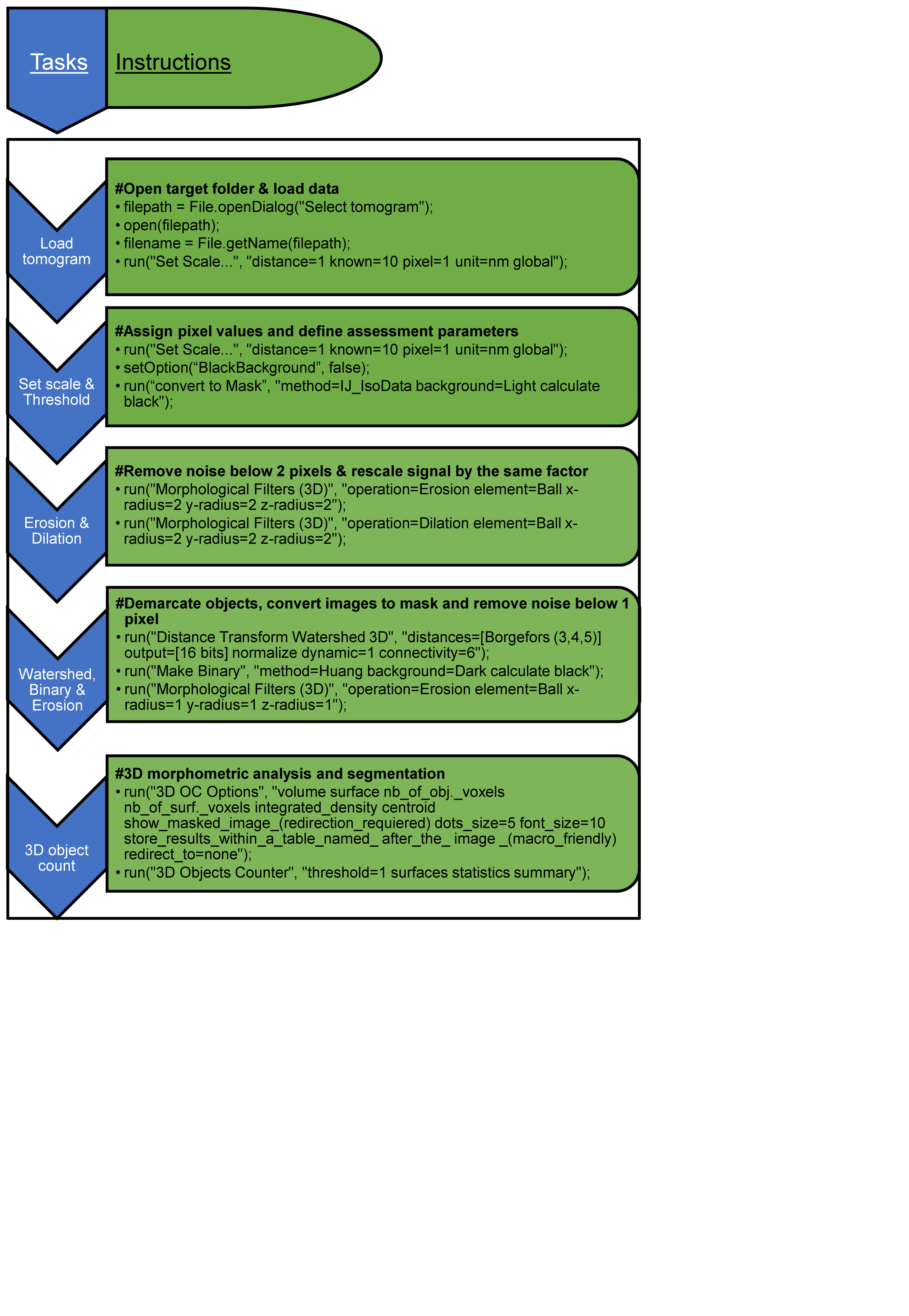

Supplement: Supplementary file 2 — FIGURE S2. Chart showing the list of instructions and the desired tasks to be carried out as contained in the SegORBS macro‐script used in automated segmentation of 3D AV datasets. The lowest level of watershed filter (level 1) was selected for proper demarcation in the event of the occurrence of joined AVs in any FOV being analysed [file JMI-284-214-s002.tiff]
